# Cone beam computed tomography assessment of the anatomical variations in mandibular lingual foramen: A strobe retrospective cross-sectional study

**DOI:** 10.1097/MD.0000000000043138

**Published:** 2025-12-26

**Authors:** Ali Ocak

**Affiliations:** a Department of Dentomaxillofacial Radiology, Faculty of Dentistry, Erzincan Binali Yildirim University, Erzincan, Türkiye.

**Keywords:** anatomical variations, anterior mandible, cone‑beam computed tomography, implant surgery, lingual foramen

## Abstract

The anterior mandible is a region that may frequently receive insufficient attention in surgical interventions; however, is crucial to recognize that this area associated with significant complications due to the presence of lingual foramina and the vascular structures passing through it. Notably, these foramina may exhibit variations in both different numbers and locations, necessitating diligent during surgical planning. The objective of this study is to evaluate the location, distribution, and prevalence of the median lingual foramen and lateral lingual foramen (LLF) as identified through cone-beam computed tomography (CBCT) in a sample drawn from the Turkish population. Measurements were taken concerning the frequency, distribution, location, and diameter (Df) of both the median lingual foramen and LLF. Additionally, the vertical distance to the inferior border of the mandible (Hinf) for each foramen, the horizontal distance (Hhor) from the deepest point of the median lingual canal and lateral lingual canal to the buccal cortical plate, as well as the lengths of the canals (Lc) were systematically recorded. The analysis revealed no statistically significant differences in the prevalence of lingual foramina or in the measurements of Df, Hinf, Hhor, and Lc when comparing male and female patients. It was observed that all CBCT scans exhibited the presence of at least 1 lingual foramen in the mandible. Notably, a significantly greater occurrence of LLFs was identified in the mandibular premolar region compared to other anatomical regions. The findings indicate a higher prevalence of mandibular lingual foramina within the Turkish population compared to previous studies. Specifically, the variations in the anatomy of mandibular lingual foramina and canals warrant careful consideration through CBCT imaging prior to surgical interventions in proximity to the inner surface and basis of the mandible, particularly in the anterior and premolar regions, to mitigate the risk of potential complications.

## 1. Introduction

The mandible is an important structure within the human skeletal system, characterized by the presence of numerous unnamed foramina that facilitate the passage of nerves and blood vessels, particularly noticeable on its internal surface.^[1]^ Acknowledgments of anatomical variations within the mandible is crucial for preventing potential complications that may arise prior to surgical interventions in this area. Comprehensive treatment planning and the execution of surgical procedures requires a deep understanding of the anatomical variations present in the anterior mandible, as this knowledge is essential for minimizing complications such as hemorrhage or nerve damage.^[2–4]^ The mandibular symphysis is often regarded as an undesirable location for dental implant placement; however, alternative regions are considered comparatively safer for surgical operations.^[5]^

The lingual foramen (LF), a small round opening located in the region of the mandibular symphysis, is referenced in the literature by various names depending on its precise anatomical context. No standardized terminology has been established for this anatomical landmark; certain studies refer to it as the lingual vascular foramen, the lateral lingual foramen or the accessory lingual foramen.^[1,2,6,7]^ The LF is predominantly categorized into 2 types based on its anatomical positioning: the median lingual foramen (MLF), which is located within the mandibular symphysis and the lateral lingual foramen (LLF), observed in a more posterior position concerning the mandibular midline.^[7]^ Notable variability exists in the characteristics of lingual foramina across individuals, with some individuals have 1 or more foramina. Moreover, the conventional radiographic detection of these foramina presents major challenges, especially in terms of measuring their number and length.^[8]^ Currently, surgical interventions in the interforaminal region of the mandible are commonplace and are generally considered safe; however, this prevailing sentiment does not rest on a precise anatomical information.^[7,9]^ The LF is crucial as it functions as a nutrient canal and also facilitates the exit of the artery formed by the anastomosis of 2 sublingual arteries.^[10,11]^

Notably, the median lingual canal houses the lingual artery, sublingual artery, branches of the mylohyoid nerve, lingual nerve, and the mandibular incisive nerve, while the lateral lingual canal accommodates a neurovascular bundle that originates from the submental artery, inferior alveolar artery as well as the inferior alveolar nerve. Injury sustained to the lingual foramen and associated canal contents can result in significant neurosensory disorders and hemorrhagic events.^[1,3]^ The close relationship between the lingual foramen and the possibility of hemorrhage during surgical procedures cannot be overstated; any damage to this structure could result fatal outcomes due to its proximity to the sublingual space.^[12]^

The MLF can be positioned superior, inferior or directly at the genial tubercle, typically aligning with the midline on the inner surface of the mandibular symphysis.^[7,13]^ Conversely, LLFs may present singularly or in multiples within the inner mandible’s lateral incisor, canine, or premolar regions.^[9]^ Radiographic techniques traditionally employed, such as periapical and panoramic imaging, frequently lack sensitivity in evaluating the presence of LLFs and MLFs, necessitating the utilization of cone-beam computed tomography (CBCT) for enhanced skeletal structure visualization in the maxillofacial region.^[14]^

The objective of this study is to assess the position, distribution and prevalence of the mandibular lingual foramina within a Turkish population sample as observed through CBCT imaging.

## 2. Methods

This retrospective cross-sectional study included patients who underwent CBCT imaging at Erzincan Oral and Dental Health Training and Research Hospital for diagnostic and therapeutic purposes between June 2022 and December 2023. All CBCT images were procured utilizing a PlanMeca ProMax® 3D Classic CBCT, with 0.2 mm section thickness (Romexis 6.2.1 R®software, Planmeca, Helsinki, Finland) set at parameters of 90 kVp and 6.3 mA, employing an 8 × 8 cm field of view (FOV), a 12.1-second acquisition time and a voxel size of 200 µm. The scans were conducted with the patient’s chin positioned in the chin cup, ensuring that the occlusal plane was horizontal; head alignment was adjusted using the scanner’s laser guidance system. The study incorporated adult patients aged 18 to 75 years. This research received ethics approval from Erzincan Binali Yildirim University School of Medicine Clinical Research Ethics Committee (24-03/06).

### 2.1. Inclusion criteria

Eighty-four scans exhibiting superior image quality that had optimal resolution were retained for the study. In all images presented in this study, consideration was taken to ensure that the entirety of corpus mandibula was adequately within FOV.

### 2.2. Exclusion criteria

Patients presenting with dental implants, impacted teeth, bone pathologies (e.g., cysts or tumors), CBCT artifacts or inadequate scans, in which the basis of the mandible extended beyond the FOV were excluded.

### 2.3. Statistical analysis

Population sampling calculations (alpha level = 0.05, precision = ±6.5%, expected prevalence = 90%, population size = 100,000) indicated a minimum sample size of 82 patients, with 84 patients (41 males and 43 females) determined as the appropriate sample size for this study. Data analysis was conducted utilizing Statistical Package for the Social Sciences 22 (IBM Statistics, SPSS, Chicago). Descriptive analysis was employed to summarize sample characteristics, while *t* tests were utilized for normally distributed data (including number, dimensions of foramina, canal length, distance to the basis and distance to the buccal cortical border of MLF and LLF). The Chi-square test was employed to compare categorical variables, with a significance threshold established at *P* < .05. Differences between the 2 independent groups were analyzed using the Mann–Whitney *U* test, a nonparametric alternative to the *t* test.

### 2.4. Assessment of CBCT images

A thin, canal-like radiolucency originating from the lingual surface of the mandible was identified as the lingual canal, and its anatomical opening was determined to be the LF.

The following parameters were quantitatively assessed for the MLFs:

1. Number of foramina.
2. Foramen localization relative to the genial tubercle (predominantly below or above).
3. Horizontal diameter of the foramen (Df).
4. Vertical distance from the lower margin of the foramen to the inferior mandible border (Hinf).
5. Horizontal distance from the canal’s deepest point to the buccal cortex of the mandible (Hhor).
6. Canal length (Lc).

For the LLFs, analogous parameters were measured:

1. Number of foramina.
2. Foramen positioning relative to specific teeth (from the lateral incisor to the premolar on both sides of the mandible).
3. Horizontal diameter of the foramen (Df).
4. Vertical distance from the lower margin of the foramen to the inferior mandible border (Hinf).
5. Horizontal distance from the canal’s deepest point to the buccal cortex of the mandible (Hhor).
6. Canal length (Lc).

**Figure 1.**
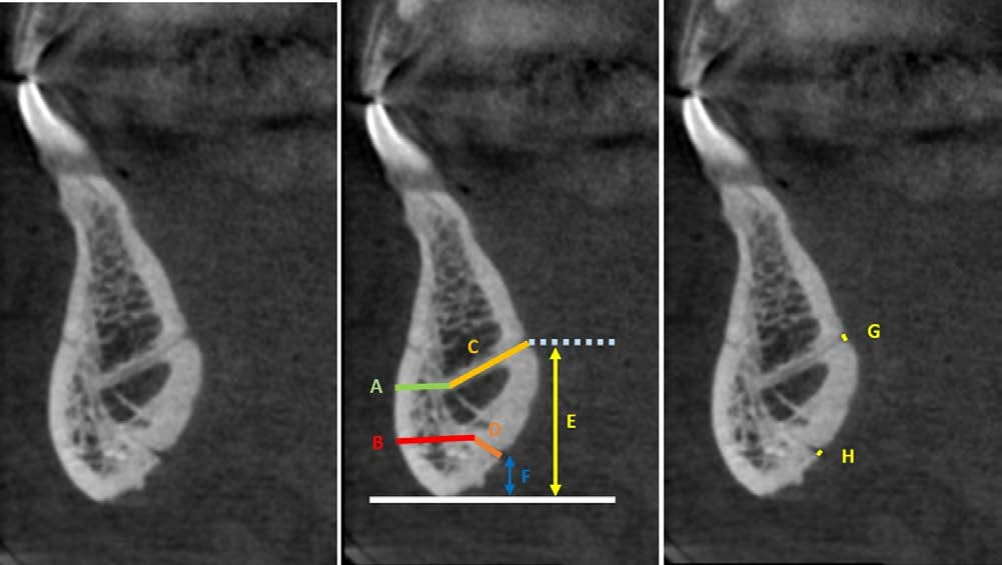
Dimensional measurements on cross-sectional mandibular CBCT images. (A and B) Distance from the deepest point of canal to the buccal cortex (Hhor). (C and D) Length of the canal (Lc). (E and F) Distance from the canal orifice to the mandibular inferior cortex level (Hinf). (G and H) Diameter of the exit orifice (Df). CBCT = cone beam computed tomography.

Moreover, the presence and number of labial foramina were recorded (Fig. 1). All CBCT images were rigorously evaluated by an oral and maxillofacial radiologist possessing a decade of clinical experience.

## 3. Results

A total of 84 CBCT scans were analyzed in this study. Among the participants, 41 were male (48.8%) and 43 were female (51.2%), with a mean age of 46.13 ± 13.89 years (47.27 ± 15.07 for males and 45.05 ± 12.74 for females), spanning an age range of 19 to 75 years. The presence of at least 1 lingual foramen was confirmed in all patients, with a maximum of 9 foramina documented. For the MLF and LLF, numbers ranged from “1 to 6” and “0 to 4,” respectively.

**Figure 2.**
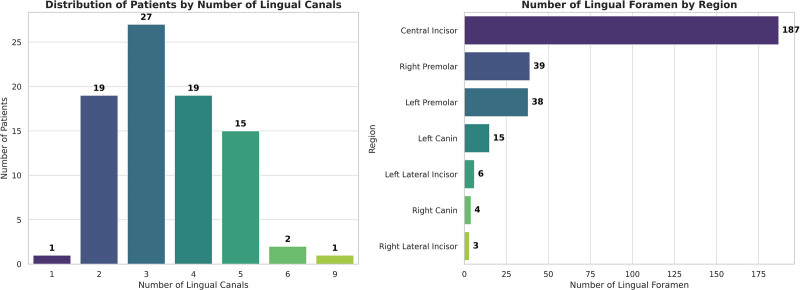
Distribution of lingual foramen numbers according to patient numbers and locations.

In line with past literature, a singular lingual foramen was detected in 1.2% of participants, while frequencies of 2 foramina were found in 22.6% of patients, 3 foramina in 32.1%, 4 foramina in 22.6%, 5 foramina in 17.9%, 6 foramina in 2.4%, and a maximum of 9 foramina was recorded for 1 individual (Fig. 2).

Both the MLF and LLF were observed in 100% and 76.19% of the patients, respectively. A total of 292 lingual foramina were recorded, comprised of 187 MLF and 105 LLF. The average number of lingual foramina per individual was 3.48 ± 1.28 (3.56 ± 1.45 for males and 3.40 ± 1.12 for females) (Table 1). No statistically significant differences were found concerning gender with respect to the number of lingual foramina (*P* = .860), median lingual foramina (*P* = .221), or lateral lingual foramina (*P* = .428), nor between patient age and the number of LF.

**Table 1:** LF, MLF, and LLF distribution between genders.

Patient gender		LF	MLF	LLF
Male	Mean	3.56	2.37	1.20
	Std. deviation	1.45	1.11	1.03
	Minimum	2	1	0
	Maximum	9	6	4
	% of total sum	50.0%	51.9%	46.7%
Female	Mean	3.40	2.09	1.30
	Std. deviation	1.12	.78	.91
	Minimum	1	1	0
	Maximum	6	4	3
	% of total sum	50.0%	48.1%	53.3%

LF = lingual foramen, LLF = lateral lingual foramen, MLF = median lingual foramen.

The average Hinf for MLF was recorded at 9.31 mm (ranging from 0.80–20.02 mm), while LLF exhibited a value of 6.80 mm (ranging from 2.61–17.00 mm). The mean Hhor for MLF was determined to be 5.63 mm (min; 2.80 mm, max; 11.41 mm), contrasting with LLF’s mean of 5.78 mm (min; 2.61 mm, max; 11.80 mm). The average canal lengths (Lc) for MLF and LLF were documented at 5.98 mm (min; 1.71 mm, max; 11.44 mm) and 5.73 mm (min; 2.00 mm, max; 11.96 mm), respectively. The average diameters (Df) of the MLF and LLF were 0.78 mm ± 0.29 mm (min; 0.24 mm, max; 1.72 mm) and 0.76 mm ± 0.25 mm (min; 0.34 mm, max; 1.63 mm), respectively. Notably, no statistically significant differences emerged in Df, Hinf, Hhor, or Lc values between male and female patients.

**Figure 3.**
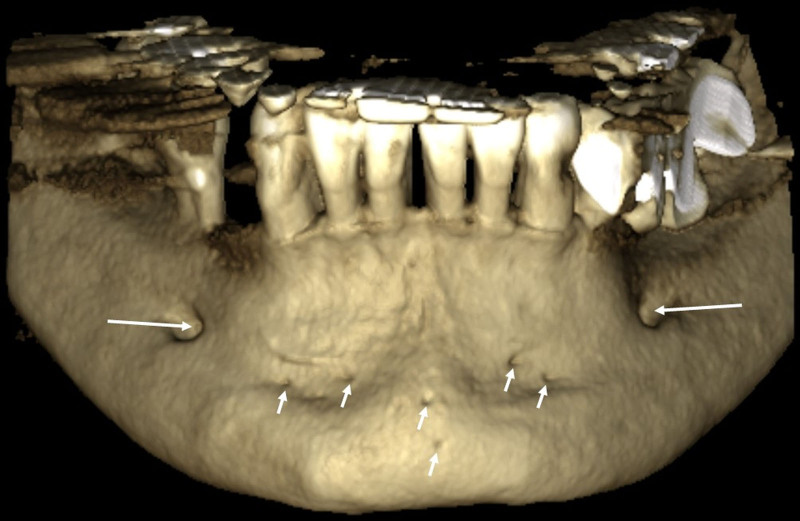
3D reconstructed CBCT image. Labial foramina (short arrows), bilateral mental foramen (long arrows). 3D = three-dimensional, CBCT = cone beam computed tomography.

**Figure 4.**
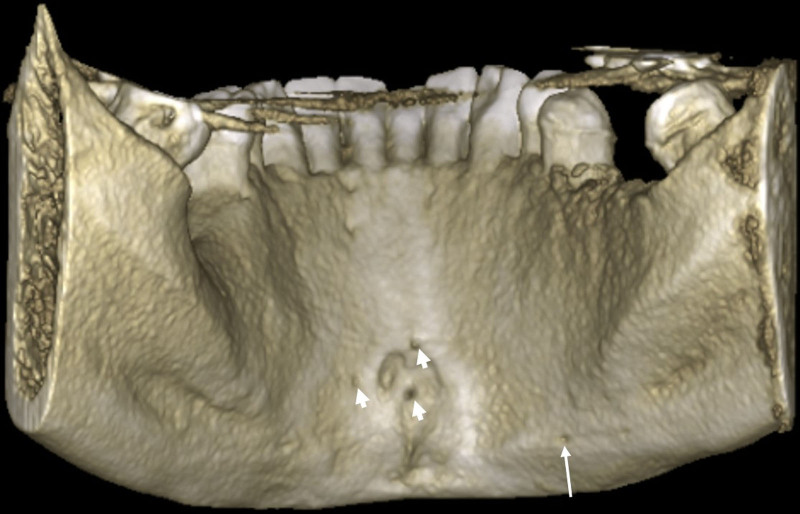
3D reconstructed CBCT image. Median lingual foramina (short arrows), lateral lingual foramen (long arrow). 3D = three-dimensional, CBCT = cone beam computed tomography.

The identified average distance (Hinf) from the MLF to the mandibular basis (9.31 mm ± 5.53) surpassed the LLF distance (6.80 mm ± 2.86). Meanwhile, other parameters (Hhor, Lc, diameter) exhibited similar values for MLF and LLF. The presence of 64 labial foramina was discerned across 36 patients, with the maximum number reaching 6 in a single individual (Figs. 3 and 4).

Within the patient cohort, the MLF was located inferior to the genial tubercle in 9 patients (10.71%), superior in 14 patients (16.67%) and both superior and inferior in 61 patients (72.62%).

## 4. Discussion

The prevalence of lingual foramina has frequently been documented at high rates, ranging from 77.90% to 100% across various studies.^[1,7,10,15–19]^ In alignment with existing literature, this study confirms that at least 1 lingual foramen was present in all evaluated patients (MLF: 100% and LLF: 76.19%). The average number of lingual foramina per individual was corroborated as 3.48 ± 1.28. Similarly, no significant differences were observed between genders regarding the frequency of lingual foramina, consistent with previous studies.^[1,7]^

Gilis et al found the location rate of MLF according to genial tubercle as 66% for superior and inferior, 26% for superior or inferior.^[15]^ In another study, Liang et al obtained these results that the presence of LFs both superior and inferior to genial tubercle were 29% of the individuals.^[10]^ Among the patients, while MLF to be positioned inferior to the genial tubercle in 9 (%10.71) patients, in 14 (%16.67) patients it was positioned superior to the genial tubercle. Also, MLF was detected both superior and inferior in 61 (%72.62) patients in this study. The rate which they observed is relatively lower according to this study and other studies may be due to the using 1 mm slice thickness and 2 mm distance apart from each slice in computed tomography (CT) images.

The distribution of lingual foramina corroborated findings from Silvestri et al, who identified 10% of patients having 1 LF, 24% with 2, and distribution percentages corresponding to 3 (30%), 4 (23%), 5 (10%), and 6 to 7 in 3%.^[1]^ He et al indicated a range of 0 to 8 foramina with the most common occurrence being 3 (24.50%).^[16]^ Scaravilli et al found the distribution of number of LF as; in 44.7% of patients had 1, 36.8% patients had 2, and 8.8% patients had 3, 9.6% of patient had any LF, also they did not report any LLF. Small-sized anatomical structures could be visualized with thin sections and small voxel sizes in CBCT and CT. They may have obtained such a result because they used 1.5 mm thickness CT sections in their studies.^[20]^ In the distribution of the number of LF according to the number of patients, similar to the literature, most of patients (32.1%) had 3 LF and 1.2% of patients had 6 or more LF was observed in this study. Prior analyses, such as Silvestri et al, reported average height measurements from the LF to the inferior mandibular border at 8.14 ± 5.25 mm, 10.6 ± 5.5 mm as noted by Liang et al, with further observations detailing heights of 11.76 mm ± 3.3 mm by Padhye et al, and 17.40 ± 7.52 mm by Yildirim et al, as 10.87 ± 2.99 mm for MLF and as 7.1 ± 4.76 mm for LLF was reported by Baghele et al.^[1,2,5,10,21]^

The Hinf of MLF was 9.31 ± 5.53 mm (min; 0.80 mm, max; 20.02 mm), this value was found as 6.80 ± 2.86 mm (min; 2.61 mm, max; 17.00 mm) for LLF in current study. A statistically significant difference was found for Hinf between MLF and LLF (*P* < .001). Accordingly, MLFs are located more superiorly, and this means that the lingual canal can be detected earlier in surgical interventions such as implant placement in this region. Padhye et al reported main horizontal distance from the deepest point of the LC to the buccal cortical border as 5.05 mm ± 1.76 mm.^[2]^ Liang et al found this value as 8.75 ± 2.28 on 132 LFs in their study.^[10]^ Silvestri et al found 6.32 ± 1.86 mm on 276 LFs in their study.^[1]^ In the current study; the Hhor of MLF was 5.63 ± 1.57 mm (min; 2.80 mm, max; 11.41 mm), Hhor of LLF was 5.78 ± 1.58 mm (min; 2.61 mm, max; 11.80 mm). There was no relationship between gender and Hhor of LFs, no statistically significant differences between MLF and LLF (*P* > .05).

Liang et al found the mean length of LCs as 6.5 ± 2.4 mm.^[10]^ This value found as 6.65 ± 4.1 mm by Gilis et al, 5.19 ± 1.63 mm by Trost et al, 6.15 ± 2.18 mm by Silvestri et al.^[1,7,15]^ These results seem to be in agreement with the current study that the average length of LC was 5.89 ± 2.19mm (median lingual canal: 5.98 ± 2.33 mm, lateral lingual canal: 5.73 ± 1.90 mm). There was no statistically significant differences between MLF and LLF (*P *> .05). Silvestri et al reported the average diameter of LFs as 0.87 ± 0.37 mm.^[1]^ Moro et al found the average diameter of MLF; 1.05 ± 0.59 mm and LLF; 0.81 ± 0.41 mm.^[17]^ This values was measured in MLF and LLF as 1.13 ± 0.29 mm and 1.10 ± 0.28 mm by Trost et al, also as 0.92 ± 0.44 mm and 0.84 ± 0.34 mm by Yildirim et al, respectively.^[5,7]^ In this study, the average Df was in MLF as 0.88 ± 0.28 mm (min; 0.24 mm, max; 1.65 mm), in LLF 0.76 ± 0.28 mm (min; 0.34 mm, max;1.63 mm). There was no statistically significant differences between MLF and LLF (*P* > .05).

It has been emphasized that lingual foramen with a diameter larger than 1 mm may cause sublingual hemorrhage during implant surgery. Trost et al found the diameters of LFs as 1.13 ± 0.29 mm and the critical size of >1 mm; 75% of MLF and 65% of LLF, in their study.^[7]^ The diameter range of LFs observed by Silvestri et al as 0.87 ± 0.37 mm and also, 30.5 % of all LFs was larger than 1 mm in their study.^[1]^ He et al found 21.23% of LFs has diameter larger than 1 mm.^[16]^ Padhye et al found that 24.9% of the subjects had LF > 1 mm in their study.^[2]^ Similarly with literature, the diameters of 18.49% (54) of LF (21.39% of MLF and 13.33% of LLF) was size of >1 mm in this study. No statistically significant difference could be found for being the diameters larger than 1 mm in between MLF and LLF.

Padhye et al observed buccal canal opening in only 8 scans (2.07%).^[2]^ Naitoh et al reported that 44% of all patients have mandibular buccal foramen in their CBCT study.^[22]^ In this study, 64 (male: 41, female: 23) buccal canals was detected. The maximum number of labial foramina was found as 6 in 1 patient. There was a statistically significant differences between genders and number of buccal foramina (*P* < .001). This result could be sourced by number of cases or may vary depending on the section thickness and the distance between sections in CBCT, compared to other studies.

In their study, Kim et al who found the ratio of LF as 58.8% and 73.20% of LFs was located in mandibular premolar region.^[23]^ Also, Krishnan et al and Wei et al reported that LLFs are mostly located below the mandibular premolar teeth 72.7% and 74.0%, respectively.^[6,14]^ In this study, agreement with the literature, LLFs are mostly located in mandibular premolar region (73.33%). A statistically significant difference was found between mandibular premolar region and lateral incisor with canin regions depending on where the LLFs are located (*P* < .001).

Today, many variety CBCT units are used with different adjustments (mA, kV, rotation degree, scan mode, voxel size, and FOV). In addition, the voxel size may vary from 0.05 to 0.6 mm. All these factors can change and affect the detailed examination of anatomical structures and create some differences between examinations. Therefore, when more details are required, it may be necessary to use higher spatial resolution examinations provided by smaller voxel sizes in suitable CBCT.^[8,24]^

## 5. Conclusion

As a conclusion of this study conveys a remarkably high prevalence of mandibular lingual foramina within the Turkish population when contrasted with findings from prior studies. Such findings may correlate with variations in the patient population sample, geographic location or more pertinently the parameters of CBCT imaging (such as voxel size and slice thickness). The anatomical variations pertinent to mandibular lingual foramina should not be overlooked before surgical approaches to the anterior mandible and premolar regions in order to mitigate potential hemorrhagic and neurologic complications.

## Author contributions

**Conceptualization:** Ali Ocak.

**Data curation:** Ali Ocak.

**Formal analysis:** Ali Ocak.

**Funding acquisition:** Ali Ocak.

**Investigation:** Ali Ocak.

**Methodology:** Ali Ocak.

**Project administration:** Ali Ocak.

**Resources:** Ali Ocak.

**Software:** Ali Ocak.

**Supervision:** Ali Ocak.

**Validation:** Ali Ocak.

**Visualization:** Ali Ocak.

**Writing – original draft:** Ali Ocak.

**Writing – review & editing:** Ali Ocak.
